# Drug Absorption Parameters Obtained Using the Isolated Perfused Rat Lung Model Are Predictive of Rat *In Vivo* Lung Absorption

**DOI:** 10.1208/s12248-020-00456-x

**Published:** 2020-05-11

**Authors:** Johanna Eriksson, Erik Sjögren, Hans Lennernäs, Helena Thörn

**Affiliations:** 1 Department of Pharmacy, Uppsala University, Box 580, SE-751 23 Uppsala, Sweden; 2 Inhalation PD Unit, Pharmaceutical Technology & Development, Operations, AstraZeneca, Pepparedsleden 1, 43183 Gothenburg, Sweden

**Keywords:** isolated perfused lung model, lung absorption, simulation, prediction, pulmonary drug delivery

## Abstract

The *ex vivo* isolated perfused rat lung (IPL) model has been demonstrated to be a useful tool during drug development for studying pulmonary drug absorption. This study aims to investigate the potential use of IPL data to predict rat *in vivo* lung absorption. Absorption parameters determined from IPL data (*ex vivo* input parameters) in combination with intravenously determined pharmacokinetic data were used in a biopharmaceutics model to predict experimental rat *in vivo* plasma concentration-time profiles and lung amount after inhalation of five different inhalation compounds. The performance of simulations using *ex vivo* input parameters was compared with simulations using *in vitro* input parameters, to determine whether and to what extent predictability could be improved by using input parameters determined from the more complex *ex vivo* model. Simulations using *ex vivo* input parameters were within twofold average difference (AAFE < 2) from experimental *in vivo* data for all compounds except one. Furthermore, simulations using *ex vivo* input parameters performed significantly better than simulations using *in vitro* input parameters in predicting *in vivo* lung absorption. It could therefore be advantageous to base predictions of drug performance on IPL data rather than on *in vitro* data during drug development to increase mechanistic understanding of pulmonary drug absorption and to better understand how different substance properties and formulations might affect *in vivo* behavior of inhalation compounds.

## INTRODUCTION

Pulmonary drug delivery is the preferred administration route for the treatment of lung diseases such as asthma, chronic obstructive lung disease, and cystic fibrosis (1). Optimal pulmonary drug delivery of locally acting active pharmaceutical ingredients (APIs) includes high local concentration, extended lung residence time, and low systemic concentration (2). These properties enhance the pharmacological effect and decrease the dosing frequency, which improves compliance and reduces the risk of systemically adverse effects (2). To ensure the efficient and successful development of inhalation drug products, improved knowledge about the pulmonary drug absorption, *i.e.*, dissolution, permeability, and tissue retention of the API in the lungs, is needed (3).

The isolated perfused rat lung (IPL) model is an *ex vivo* absorption model that allows parameters like epithelial permeability, solubility in epithelial lining fluid (ELF), dissolution rate, and particle wetting as well as tissue retention to be determined by computational analysis (4,5). The advantages of the IPL model over *in vivo* inhalation studies is that the lung-delivered dose can be closely controlled without passing the nose and the lung deposited dose can be measured directly by mass balance calculations (6,7). In addition, the effects of systemic disposition are excluded in the IPL model, because the pulmonary drug absorption is measured directly in the pulmonary vein. A physiological difference between the IPL model and the *in vivo* situation is that the systemic circulation, which supplies the bronchial region with blood, is not perfused in the IPL model (8). However, the drug absorption rate for solutes has previously been shown to correlate well between the IPL model and *in vivo* studies, which suggest that parameters obtained from the IPL model are potentially *in vivo* predictive (9). These advantages suggest that the IPL model may offer better opportunities than the more complex *in vivo* method for investigating drug absorption rate and mechanisms for solutes and different inhalation formulations.

Physiologically based biopharmaceutics (PBB) models are used to mechanistically describe absorption processes and can be used to simulate and predict drug absorption (4,5,10). These models require various drug absorption properties as input parameters, which can be either calculated from physicochemical properties or obtained from experimental measurements. For PBB models of inhaled drugs, lung absorption input parameters are commonly based on *in vitro* measurements (3). For example, permeability can be measured with cell monolayer models, tissue retention can be measured with tissue slices, and the dissolution rate can be based on solubility measures (11–13). *In vitro* measurements can be performed on a large scale while remaining cost-effective and are therefore well suited for predictions early in drug development (7). Some studies have shown that *in vitro–*based predictions can perform with high accuracy in predicting both *in vivo* and clinical plasma concentrations (14,15). However, there are still knowledge gaps in the use of input parameters in pulmonary absorption predictions (3). Absorption input parameters obtained from a more physiologically relevant experimental model like the IPL, might improve the predictive performance of PBB models, and also allow key factors relevant specifically to pulmonary absorption input parameters to be studied. Especially in later stages of drug development, the IPL can be useful in providing absorption data of for example different formulations.

The primary objective of this study was to investigate the potential use of IPL data to predict rat *in vivo* lung absorption. Absorption parameters determined from IPL data (*ex vivo* input parameters) were used in combination with intravenously determined pharmacokinetic data in the PBB model LungSim to predict experimental rat *in vivo* plasma concentration-time profiles and retained lung amount after inhalation of five different inhalation compounds. Simulations that used *ex vivo* input parameters were compared with simulations that used *in vitro* input parameters, to examine whether predictability improved when using input parameters determined from the more complex model.

## METHODS

### Study Drugs

Five study drugs (AZD5423, fluticasone furoate (FF), fluticasone propionate (FP), salbutamol, and salmeterol) were chosen based on their range of physicochemical properties and availability of data from the IPL model and intravenous (i.v.) and pulmonary administration of the drugs (Table I). For salbutamol and salmeterol, available inhalation data were based on studies with solutions, giving the opportunity to investigate pulmonary drug absorption without the effect of dissolution. For the low solubility APIs, AZD5423, FF, and FP, available inhalation data were from studies using suspensions; thus, the effect of dissolution could be examined.

**Table I:** Physicochemical Properties of the Study Drugs. All Values Were Obtained from the Chemical Library MicroSource US Drugs Found in the Database ZINC (29)

API	MW (g/mol)	cLogD	cLogP	HBD	HBA	tPSA	Net charge at pH 7.4	NRB
AZD5423	487	3.5	5.1	1	6	65	0	8
Fluticasone furoate	539	3.4	4.9	1	6	93	0	6
Fluticasone propionate	501	3.1	4.6	1	5	81	0	6
Salbutamol	240	− 1.5	1.4	5	4	77	1	5
Salmeterol	417	1.9	3.9	5	5	87	1	16

*API*, active pharmaceutical ingredient; *MW*, molecular weight; *cLogD*, logarithm of the calculated octanol/water partitioning coefficient at pH 7.4, calculated with ACD/ChemSketch® (Berkshire, UK); *cLogP*, logarithm of the calculated octanol/water partitioning coefficient; *HBD*, number of hydrogen bond donors; *HBA*, number of hydrogen bond acceptors; *tPSA*, topological polar surface area; *NRB*, number of rotatable bonds

### Previously Obtained Experimental Rat IPL Data and Developed PBB Model

By applying a physiologically based biopharmaceutics (PBB) model and analyzing data obtained from the isolated perfused lung (IPL) experiments, *ex vivo* input parameters (permeability, solubility, and tissue retention) were obtained for the LungSim model. The following sections describe these data and methods, which were obtained and developed in previous work (4,5).

#### Experimental Rat IPL Data

Experimental rat IPL data were obtained from previous studies in which solutions and suspensions of the investigated APIs were administered (4,5). Briefly, the heart and lungs were isolated from the rat and put in a humidified chamber. A buffer containing albumin and glucose were used to single-pass perfuse the pulmonary circulation at a rate of 20 mL/min. The lungs were ventilated at a rate of 75 breaths per minute. The solutions (0.03 mg/mL or 0.1 mg/mL) and suspensions (1 mg/mL) were nebulized to the IPL model for 60 and 30 s, respectively. Over the course of the experiment (up to 90 min), samples were collected with an automatic sampler at predetermined time points. At the end of the experiment, the lungs were cut from the heart and trachea and were frozen for further quantitative analysis of the parent drug with the LC-MS/MS method. The lung deposited dose of API was calculated as cumulative amount in perfusate and the amount in the lungs at the end of the experiment.

#### IPL PBB Model

A previously developed PBB model was applied to mechanistically describe the rate and extent of pulmonary absorption based on experimental IPL data and to estimate biopharmaceutics parameters relevant to pulmonary absorption such as wetting, dissolution, permeability, and tissue retention (4,5). Briefly, the PBB model has two regionally (alveolar and tracheobronchial) specific descriptions of the deposited dose, the epithelial lining fluid (ELF), and the intracellular and vascular spaces in the lung tissue, including intra- and extracellular drug binding sites. An alveolar:tracheobronchial (Al:Tb) region lung deposition ratio of 3:2 was applied for both the solutions and suspensions (4,5). The following equations (Eqs. 1–3) were used to describe the absorption process in the lungs:

1 $$ \mathrm{dissolution}\ \mathrm{rate}:{k}_{\mathrm{diss}}=\frac{k\times D\times {A}^{\raisebox{1ex}{$2$}\!\left/ \!\raisebox{-1ex}{$3$}\right.}\times \left( Cs-C\right)}{h\times {r}^2} $$

where *k*_diss_ is the rate of dissolution, *k* is a constant, *D* is the diffusion coefficient, *A* is the amount, *Cs* is the solubility in ELF, *C* is the concentration in ELF, *h* is the thickness of diffusion layer, and *r* is the radius of the particle. The particle radius was divided into eight bins in each region to account for the particle size distribution. The equation for the dissolution rate also takes into account the shrinking of particles, where *A* is raised by 2/3.

2 $$ \mathrm{Drug}\ \mathrm{transport}\ \mathrm{across}\ \mathrm{the}\ \mathrm{epithelial}\ \mathrm{membrane}:\frac{dA}{dt}={P}_{\mathrm{mem}}\times \frac{A}{V}\times {\mathrm{SA}}_{\mathrm{mem}} $$

where *P*_mem_ is the membrane permeability, *A* and *V* are the amount and volume related to the donor compartment, respectively, and *SA*_mem_ is the surface area of the membrane. The effective pulmonary permeability (*P*_eff_) is described as *P*_eff_ = *P*_mem_/2.

3 $$ \mathrm{Drug}\ \mathrm{transport}\ \mathrm{from}\ \mathrm{the}\ \mathrm{vascular}\ \mathrm{space}\ \mathrm{to}\ \mathrm{the}\ \mathrm{perfusate}:\frac{dA}{dt}=\frac{Q}{V_{\mathrm{vasc}}}\times {A}_{\mathrm{vasc}} $$

where *Q* is the single-pass perfusion rate (20 mL/min) and *V*_vasc_ and *A*_vasc_ are the volume and amount of drug related to the vascular compartment.

The intra- and extracellular tissue retention were described with the rate constants *k*_in_ and *k*_out_.

(For further information about the IPL PBB model and derivations of the above equations, see references 4 and 5.)

### LungSim Model

**Fig. 1:**
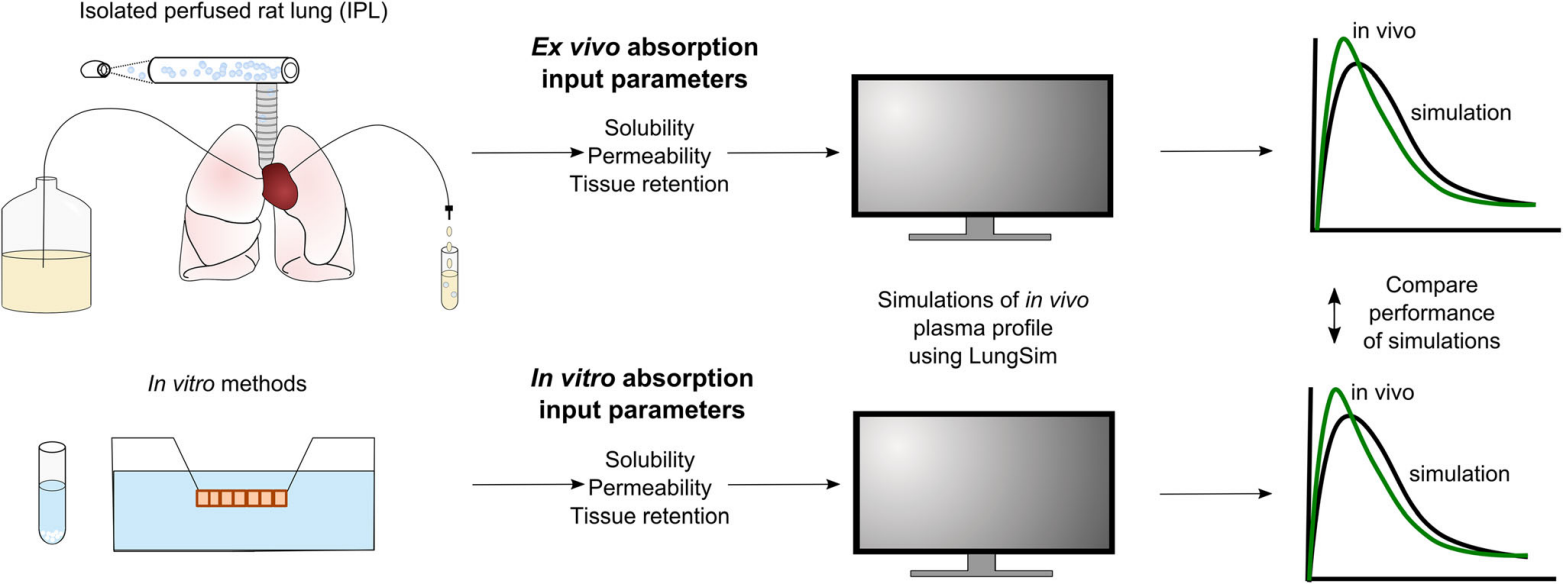
Schematic illustration of the outline of this study

LungSim was developed by AstraZeneca as an in-house PBB model for simulations of lung and plasma concentrations after drug administration via the pulmonary route (15,16). LungSim is an extension of GI-Sim, a previously developed biopharmaceutics tool for predictions of gastrointestinal drug absorption (17). LungSim has a lung deposition model and a lung absorption model, but because the focus of this work is on absorption input parameters, lung deposition patterns were obtained from the literature instead of estimated using the deposition model in LungSim. Absorption parameters obtained from IPL (*ex vivo* input parameters) or *in vitro* models (*in vitro* input parameters) were combined with systemic distribution and elimination for simulations of plasma concentrations and lung amounts (Fig. 1). Thus, the absorption input parameters (*P*_eff_, solubility and tissue retention) differed between the two settings (*ex vivo* and *in vitro*) while formulation-specific parameters (particle size and deposition pattern) and pharmacokinetic parameters (plasma clearance, the volume of distribution, fraction unbound in plasma and distribution parameters) applied in the LungSim model were the same for both settings (Table II).

**Table II:** *In Vitro* and *Ex Vivo* Absorption Parameters, Formulation, and Pharmacokinetic (PK) Parameters Used in the Simulations

	AZD5423		FF		FP		Salbutamol		Salmeterol	
	*In vitro*	*Ex vivo*	*In vitro*	*Ex vivo*	*In vitro*	*Ex vivo*	*In vitro*	*Ex vivo*	*In vitro*	*Ex vivo*
Absorption parameters										
*P*_eff_ (×10^−4^ cm/s)	0.690	0.0024	1.98	0.0029	2.14	0.0041	0.04	0.0015	0.31	0.00074
Scaled *P*_eff_	Yes	No	Yes	No	Yes	No	Yes	No	Yes	No
*f*_u,tissue_ (%)	0.06	7	0.15	5	0.47	0.1	45	1	0.12	22
Dynamic distribution (min)	n.a.	*k*_in_ 0.17 *k*_out_ 0.014	n.a.	*k*_in_ 0.17 *k*_out_ 0.009	n.a.	*k*_in_ 6.4 × 10^−5^ *k*_out_ 6.3 × 10^−8^	n.a.	*k*_in_ 6.6 × 10^−5^ *k*_out_ 6.4 × 10^−7^	n.a.	*k*_in_ 0.056 *k*_out_ 0.016
Solubility (μmol/L)	0.6	n.a.	0.036	0.24	0.18	0.32	n.a.	n.a.	n.a.	n.a.
Formulation parameters										
Droplet size (μm) (span)	1.83 (2.6)		1.83 (2.6)		1.83 (2.6)		n.a.		n.a.	
Particle size (μm) (span)	1.29 (3.3)		2.13 (1.1)		1.97 (1.3)		n.a.		n.a.	
Lung-delivered particle size (μm) (span)	0.34 (1.6)		0.95 (0.2)		0.90 (0.3)		n.a.		n.a.	
Al:Tb ratio	50:50		50:50		50:50		30:70		30:70	
LDD (μg/kg)	24		23		25		20		20	
PK parameters										
*f*_u,plasma_ (%)	0.1		0.5		1.6		65		7.5	
*V*_d_ (L/kg)	2.72		3.16		3.28		0.480		2.21	
CL (mL/min/kg)	39.3		59.5		73.8		38.5		151	
*k*_12_ (h)	0.564		0.334		0.329		6.62		16.7	
*k*_21_ (h)	0.141		0.432		0.352		2.49		1.11	

*P*_*eff*_, effective permeability; *f*_*u,tissue*_, fraction unbound tissue; *Al:Tb*, alveolar:tracheobronchial; *LDD*, lung-delivered dose; *F*_*u,plasma*_, fraction unbound plasma; *Vd*, volume of distribution; *CL*, plasma clearance; *k*_*12*_ *and k*_*21*_, distribution rate constants; *n.a.*, not applicable

### *Ex Vivo* Input Parameters

The IPL PBB model is similar to the LungSim PBB model, but has a few key differences:

- The applied volumes and areas of the alveolar and tracheobronchial region are different between the two models.
- The *P*_eff_ value is scaled based on the thickness of the epithelium in the LungSim model, resulting in regional values for *P*_eff_. In the IPL PBB model, the *P*_eff_ value is not scaled (*i.e*., the same value was applied in the alveolar and tracheobronchial region).
- The vascular space is not divided into alveolar and a tracheobronchial region as it is in the IPL PBB model (4).
- The dissolution rate is slightly differently described in LungSim than the IPL PBB model.
- Removal of drug particles from ELF by mucociliary clearance exists in LungSim but not in the IPL PBB model.

These differences needed to be assessed to ensure that the *ex vivo* input parameters represented the absorption in the experimental IPL model when used in the LungSim model.

To correct for these differences, *P*_eff_ and tissue retention (*k*_in_ and *k*_out_) were re-estimated using the previously developed IPL PBB model but applying the same volumes and surface area as in LungSim (Phoenix® WinNonlin® 8.1, Certara USA, NJ, USA) (4). The difference in the division of vascular space was not corrected for because it did not affect the absorption rate (data not shown). (To account for parameter specific differences, see the sections below.)

#### Permeability

The re-estimated pulmonary *P*_eff_ was used as an input value for lung *P*_eff_ (4) (Table II). In opposition to default settings, no scaling of the *P*_eff_ value between alveolar and bronchial regions was adopted in the LungSim model, by analogy with the modeling strategy for estimation of this parameter.

#### Tissue Retention

The re-estimated values for *k*_in_ and *k*_out_ were used as input values for tissue retention in LungSim (4) (Table II). These values describe tissue retention as a dynamic process, where *k*_in_ is the rate of distribution into the tissue and *k*_out_ is the rate of distribution out of the tissue. The LungSim model requires a *f*_u,tissue_ factor, calculated as:

4 $$ {f}_{\mathrm{u},\mathrm{tissue}}=\frac{1}{1-\left(\raisebox{1ex}{${k}_{\mathrm{in}}$}\!\left/ \!\raisebox{-1ex}{${k}_{\mathrm{out}}$}\right.\right)} $$

#### Solubility

As mentioned, the dissolution is described slightly differently in the IPL PBB model and the LungSim model. LungSim allows estimation of solubility based on a dissolution concentration-time profile, which was performed to ensure a correct *ex vivo* input value for solubility. Dissolution profiles from the IPL experiments were obtained by applying the IPL PBB model.

As previously reported, the absorption rate in the IPL model for the suspension and solution of AZD5423 was the same, indicating that dissolution was not rate-limiting for the suspension (with a similar particle size distribution as used in the *in vivo* validation data) and a dissolution profile not possible to obtain (5). Therefore, AZD5423 was in this study simulated as a solution in LungSim even if administered as a suspension.

### *In Vitro* Input Parameters

#### Permeability

Intestinal *P*_eff_ was used as the *in vitro* input parameter for pulmonary *P*_eff_ (Table II). The intestinal *P*_eff_ was calculated from a Caco-2 apparent permeability (*P*_app_) − intestinal *P*_eff_ correlation (intestinal *P*_eff_ = 1.8 *P*_app_ + 1.06, human UWL thickness = 8.6 × 10^−5^, Caco-2 UWL thickness 7.5 × 10^−4^ (17,18)). The Caco-2 measurements were performed with major drug influx and efflux transporters inhibited. Values for Caco-2 *P*_app_ were obtained from Eriksson *et al.* (4). The scaling of pulmonary *P*_eff_ in LungSim was kept as default for the simulations using *in vitro* input parameters.

#### Tissue Retention

The drug fraction unbound in the lung (*f*_u,tissue_) was used as the parameter for describing tissue retention and was obtained using volume unbound (*V*_u,lung_) measured in lung slices as described by Bäckström *et al.* (11), where *f*_u,tissue_ = 1/*V*_u,lung_× *V*_u,lung_ values were obtained from AstraZeneca in-house database (Table II). The tissue distribution settings in LungSim were set to “equilibrium” to accommodate for this input parameter, and so distribution time dependencies were not evaluated in these simulations.

#### Solubility

The input value for *in vitro* solubility was the solubility in phosphate buffer at pH 7.4, as reported by Eriksson *et al.* (5) (Table II).

### Pharmacokinetic Input Parameters

Intravenous plasma concentration-time profiles for all APIs were obtained from AstraZeneca in-house database. The in-house studies were approved by the local ethics committee. The i.v. bolus doses administered to the rats were 0.924 and 0.802 μmol/kg for AZD5423, 0.943 and 0.922 μmol/kg for FF, 1.03 and 0.949 μmol/kg for FP, 4.48 μmol/kg for salbutamol (three replicates) and 2.43 μmol/kg for salmeterol (two replicates). Plasma samples were retrieved at predetermined time points over 24 h for AZD5423, FF, and FP, over 6 h for salbutamol and over 12 h for salmeterol. The i.v. profiles were analyzed by applying a two-compartment model to obtain pharmacokinetic parameters describing the systemic distribution and elimination, *i.e*., volume of distribution (*V*_d_), rate of distribution (*k*_12_ and *k*_21_), and plasma clearance (CL) (Phoenix® WinNonLin® 8.1, Certara USA, NJ, USA) (Table II). Estimated pharmacokinetic parameters were used to describe systemic distribution and elimination in the LungSim model.

### Description of *In Vivo* Validation Dataset

The *in vivo* validation dataset was obtained from AstraZeneca in-house database and included both nose-only inhalation and intratracheal instillation data. The in-house studies were approved by the local ethics committee.

#### *In Vivo* Nose-Only Inhalation Data

AZD5423, FF, and FP were administered as nose-only inhalation to rats. The lung-delivered dose was calculated to be between 23 and 25 μg/kg for the APIs. The APIs were nebulized as suspensions over 30 min. Measurements of droplet and particle size distributions were performed in-house at AstraZeneca (Table II). The median particle size and span of the particles were adjusted to represent the lung-delivered particle size distribution by only considering the particles ≤ 1 μm to deposit in the lungs (Table II). This assumption was based on the study by Schmid et al. (19), where particles ≥ 1 μm to a high extent deposit in the nose and extrathoracic region. The regional deposition (Al:Tb ratio) for nose-only inhalation was assumed to be 50:50 (20). Both plasma concentration and lung amount were available for the inhalation data.

#### *In Vivo* Intratracheal Instillation Data

Lung-delivered doses of 20 μg/kg of salbutamol and salmeterol were administered to rats as an intratracheal instillation (i.t.) (LDD in Table II). The APIs were administered as solutions. The Al:Tb ratio for i.t. administration was assumed to be 30:70 (21). Only plasma concentration was available for the i.t. data.

### Simulations

Simulations for each API were performed in LungSim with the *ex vivo* and *in vitro* input parameters summarized in Table II. For each API, additional simulations were performed using ex vivo input parameters in which the bronchial permeability values were doubled. This was done to compensate for the possibility of lower bronchial absorption in the IPL model than *in vivo*, because the tracheobronchial region is not perfused in the IPL model. Because deposition pattern is an uncertain parameter in predictions of lung absorption, higher Al:Tb ratios were also applied (80:20 for AZD5423, FF, and FP and 50:50 for salbutamol and salmeterol) to test the effect.

### Statistical Evaluation

The similarity between simulated and experimental data was evaluated by comparing the area under the curve (AUC), maximum concentration (*C*_max_), time when *C*_max_ occurred (*t*_max_), absolute average fold error (AAFE), and average fold error (AFE). AUC, *C*_max_, and *t*_max_ were calculated using GraphPad Prism 8® (GraphPad Software, San Diego, USA). AAFE and AFE were defined as follows:

5 $$ \mathrm{AAFE}={10}^{\sum \mid \log \left(\frac{sim}{expe}\right)\mid /N} $$

6 $$ \mathrm{AFE}={10}^{\sum \log \left(\frac{sim}{expe}\right)/N} $$

where *sim* is the simulated data, *expe* is the experimental data, and *N* is the number of data points included. An AAFE value of 1 indicates a perfect agreement between simulated and experimental data, while a value of 2 indicates an average twofold difference between the compared data. AAFE can therefore be used to evaluate the difference between two datasets and AFE evaluates whether the simulation over- or underestimates the experimental data.

## RESULTS

### Simulations Using *Ex Vivo* Input Parameters

**Fig. 2:**
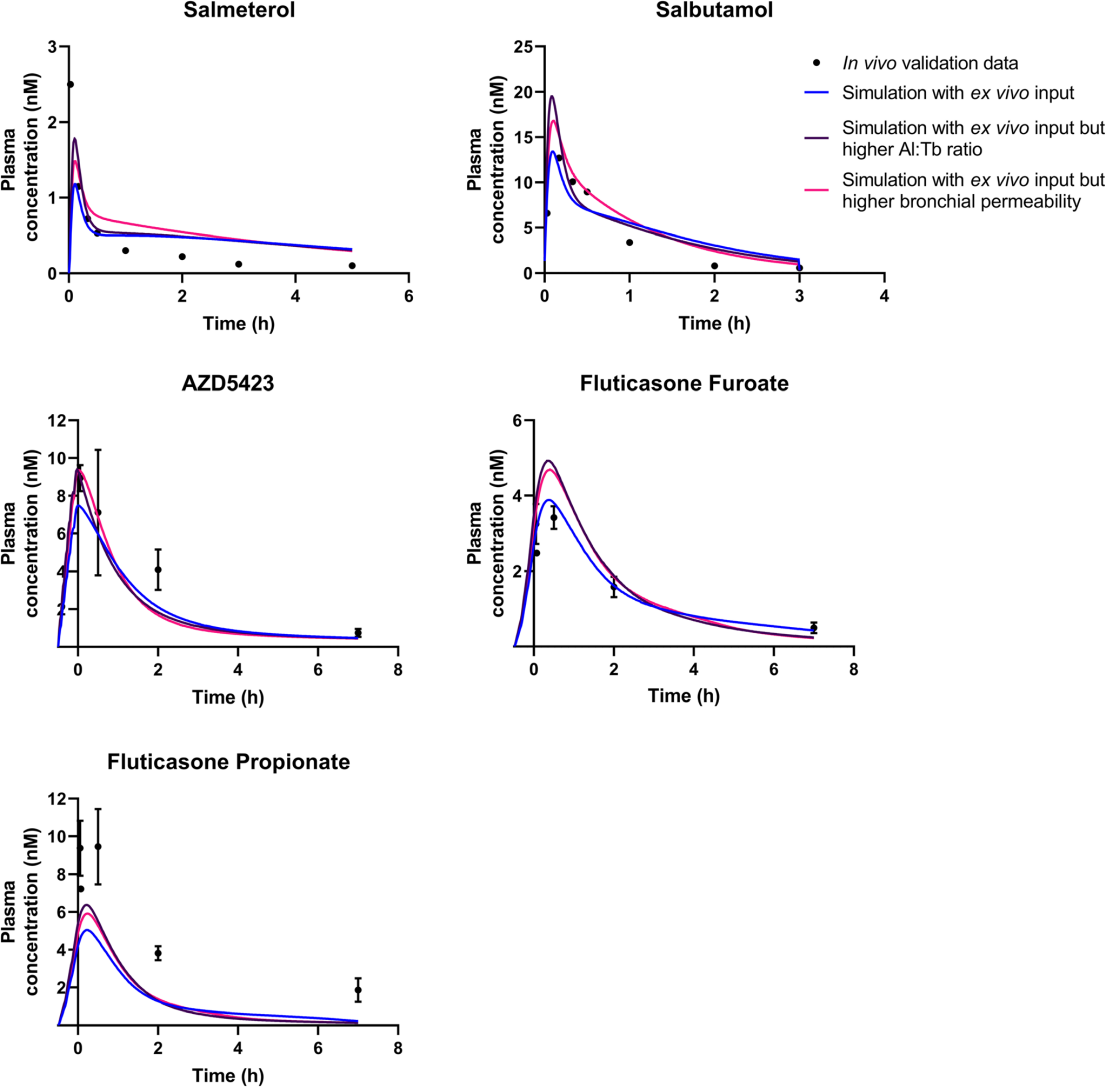
Experimental rat *in vivo* plasma concentration (dots) and plasma concentration simulated using *ex vivo* input parameters (lines)

**Fig. 3:**
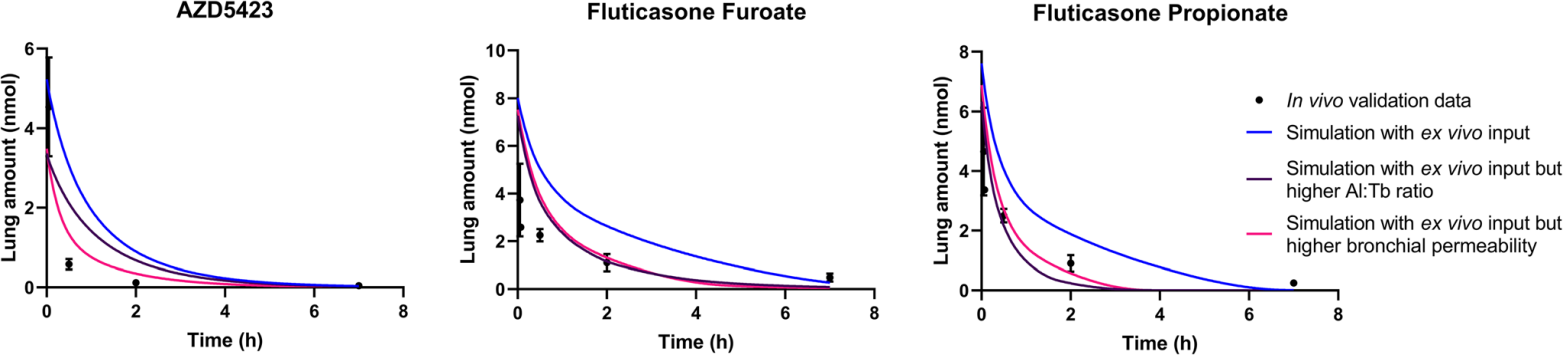
Experimental rat *in vivo* lung amount (dots) and lung amount simulated using *ex vivo* input parameters (lines)

**Fig. 4:**
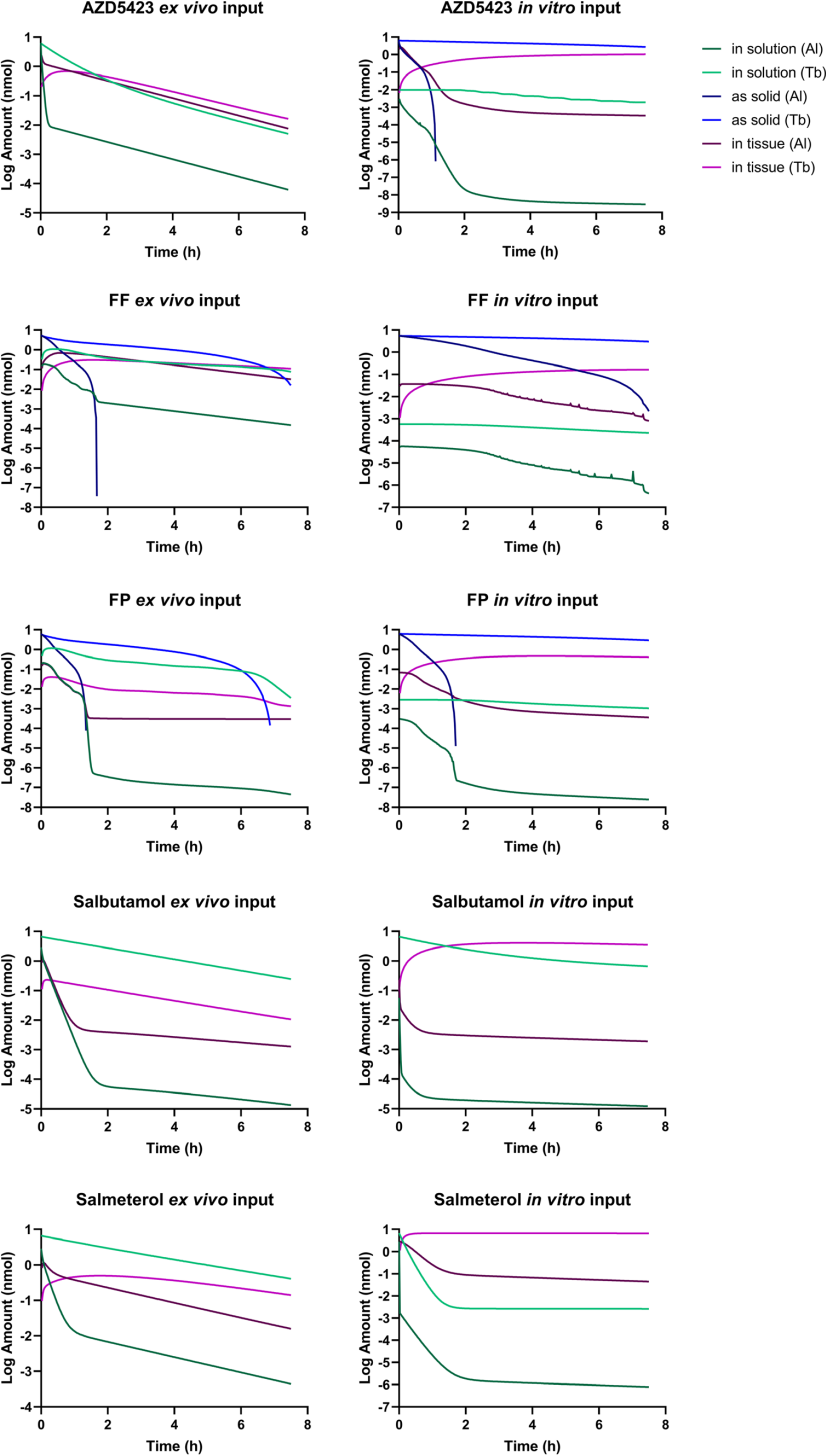
Simulated lung amount in each compartment of the lung (as solid, ELF, and in tissue) were plotted over time for both regions (alveolar (Al) and tracheobronchial (Tb)) for simulations using *ex vivo* or *in vitro* input parameters

The simulations using *ex vivo* input parameters were within twofold average difference (AAFE < 2) from the experimental *in vivo* data for all APIs except FP (Fig. 2, Table III). In general, the simulations had a lower *C*_max_ than the experimental *in vivo* data (Table III). The simulated lung amount was higher than the experimental lung amount for all APIs (Fig. 3). The simulations with higher bronchial permeabilities and a higher Al:Tb ratio gave a comparatively similar plasma concentration-time profile (difference in *t*_max_ < 0.1 h and *C*_max_ < 20%) (Fig. 2, Table III). With these modified simulation settings, the predicted *C*_max_ improved for AZD5423, FP, and salmeterol, and AAFE improved for AZD5423, FP, and salbutamol compared with the initial simulation settings (Table III). In addition, the modified simulation settings improved the predictions of lung amount for all APIs (Fig. 3). Absorption in the alveolar region was in general rapid (> 90% of drug absorbed within 1 h), while it was slower in the tracheobronchial region (Fig. 4). AZD5423 permeated the epithelial membrane and was then retained in the tissue (the slope of the amount in tissue over time is less steep than the slope of the amount in solution over time, Fig. 4). FF and FP dissolved slowly in the tracheobronchial region; FF was retained in the tissue after permeation while FP was not (Fig. 4). For salbutamol, permeation was the rate-limiting step, while for salmeterol, tissue retention was rate-limiting (Fig. 4).

**Table III:** Statistical Evaluation Including Time to Maximum Concentration (*t*_max_), Maximum Concentration (*C*_max_), Area Under the Curve (AUC), Absolute Average Fold Error (AAFE), and Average Fold Error (AFE) for The Experimental *In Vivo* Data and the Simulations (sim.) Performed

Statistical parameter	Experimental *in vivo* data	Sim*. ex vivo* input	Sim. higher bronch. perm.	Sim. higher Al:Tb ratio	Sim. *in vitro* input
AZD5423					
*t*_max_ (h)	0.050	0.00	0.025	− 0.025	0.12
*C*_max_ (nM)	8.93	7.48	9.36	9.39	5.09
AUC (nM × h)	24.1	15.5	15.8	15.7	8.00
AAFE	n.a.	1.45	1.45	1.44	2.74
AFE	n.a.	0.69	0.71	0.70	0.37
Fluticasone furoate					
*t*_max_ (h)	0.50	0.35	0.40	0.33	1.38
*C*_max_ (nM)	3.42	3.88	4.69	4.92	1.44
AUC (nM × h)	10.3	10.3	11.2	11.3	5.64
AAFE	n.a.	1.12	1.42	1.46	2.70
AFE	n.a.	1.03	1.04	1.10	0.37
Fluticasone propionate					
*t*_max_ (h)	0.50	0.20	0.23	0.20	0.25
*C*_max_ (nM)	9.45	5.04	5.91	6.37	3.51
AUC (nM × h)	27.9	10.1	10.5	10.7	5.67
AAFE	n.a.	2.76	2.73	2.66	4.66
AFE	n.a.	0.36	0.37	0.38	0.21
Salbutamol					
*t*_max_ (h)	0.17	0.09	0.10	0.08	0.00
*C*_max_ (nM)	12.7	13.4	16.8	19.5	20.0
AUC (nM × h)	10.7	14.2	15.3	14.7	7.54
AAFE	n.a.	1.59	1.56	1.66	2.88
AFE	n.a.	1.15	1.29	1.26	0.56
Salmeterol					
*t*_max_ (h)	0.03	0.10	0.10	0.10	0.20
*C*_max_ (nM)	2.50	1.18	1.48	1.78	0.62
AUC (nM × h)	1.37	2.32	2.68	2.48	0.873
AAFE	n.a.	1.99	2.14	1.94	1.95
AFE	n.a.	1.27	1.56	1.47	0.52

*Al:Tb*, alveolar:tracheobronchial; *n.a*., not applicable

### Simulations Using *In Vitro* Input Parameters

**Fig. 5:**
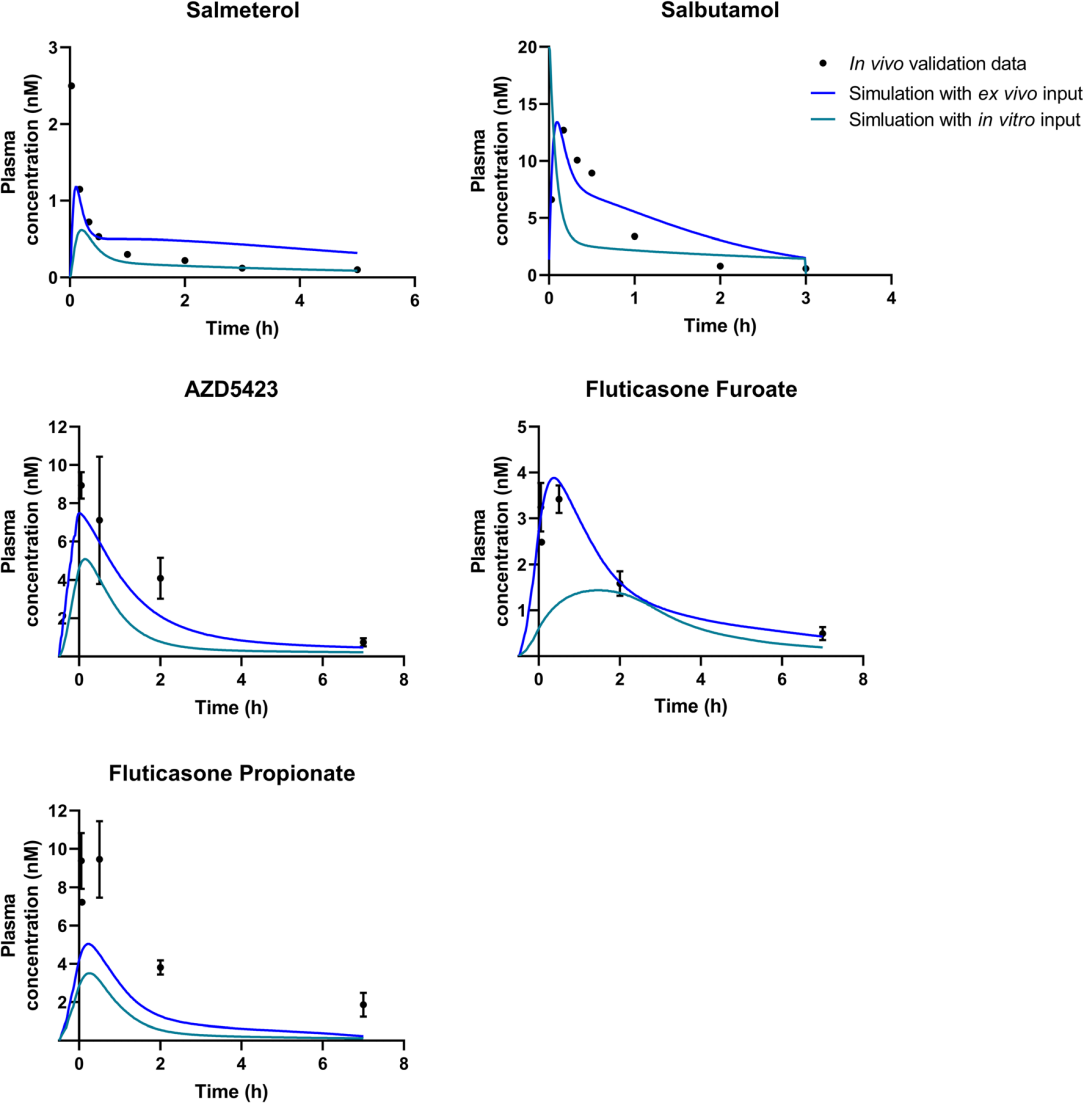
Experimental rat *in vivo* plasma concentration (dots) and plasma concentration simulated using *ex vivo* or *in vitro* input parameters (lines)

**Fig. 6:**
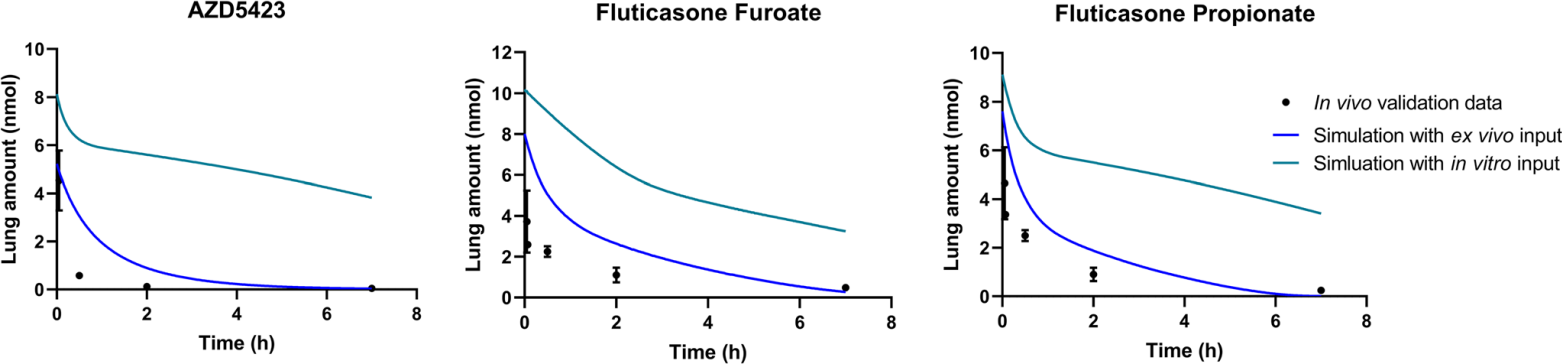
Experimental rat *in vivo* lung amount (dots) and lung amount simulated using *ex vivo* or *in vitro* input parameters (lines)

The simulations using *in vitro* absorption parameters had AAFEs > 2 compared with experimental *in vivo* data for all APIs except salmeterol (Table III). In general, the simulations under predicted the rate of pulmonary absorption (Figs. 5 and 6). The *in vitro* simulations were less accurate in predicting *C*_max_ than the simulations using *ex vivo* input parameters for all APIs (Fig. 5) and had a higher AAFE for all APIs except salmeterol (where the two AAFEs were nearly equal, Table III). For salmeterol, the *ex vivo* input parameters predicted the *C*_max_ better, while the *in vitro* input parameters gave a better prediction for the later time points of plasma concentration (Fig. 5). The dissolution rate and tissue retention in the tracheobronchial region were the rate-limiting steps for the absorption of AZD5423, FF, and FP (Fig. 4). For salbutamol and salmeterol, tissue retention in the tracheobronchial region was rate-limiting (Fig. 4).

## DISCUSSION

Lung-specific absorption parameters obtained via the IPL model were used to simulate rat *in vivo* plasma concentration-time profile and lung amount for five different inhalation compounds. Simulations were within 2-fold absolute average error (AAFE) of the experimental *in vivo* rat data, with the exception of fluticasone propionate, indicating that drug absorption parameters obtained from the IPL model are predictive of *in vivo* lung absorption. Simulations using *in vitro* input parameters were compared with simulations using *ex vivo* input parameters, and those based on *ex vivo* parameters were significantly more accurate (as indicated by lower AAFE values) for the investigated APIs (except for salmeterol where the difference in AAFE was insignificant). These results demonstrate the advantage of the IPL method over *in vitro* methods for determining input parameters for predictions of *in vivo* plasma concentration-time profile and lung amount. In contrast to *in vitro* models, the IPL model resembles the *in vivo* dissolution better because it offers physiologically relevant volume, fluid composition, and sink/non-sink conditions. IPL also resembles *in vivo* permeability better because the epithelial membrane is the same as would be found in an *in vivo* experiment; for similar reasons, IPL resembles *in vivo* tissue retention better than *in vitro* because it offers the same volume, tissue composition, and dynamic binding processes. In addition, IPL includes the diversity and complexity of lung structure, including differences in the abovementioned parameters between the alveolar and tracheobronchial regions.

IPL is an advanced *ex vivo* model closely mimicking the *in vivo* lung physiology because it includes continuous lung breaths and the lung is perfused. One significant difference between IPL and *in vivo* experiments is that the bronchial region is not perfused in IPL; however, absorption parameters obtained from IPL have been shown to be predictive of *in vivo* lung absorption. Because the amount absorbed in IPL has reached near-complete absorption in several studies, it has been suggested that drug can be absorbed from the bronchial region, despite this region is not being perfused (4,8). Such a result might be explained by drug transport from the bronchial region through anastomoses and/or diffusion to the alveolar capillaries (22). Increasing permeability in the tracheobronchial region for the simulations improved predictions (lower AAFE) for three out of five investigated APIs. Based on that result, it seems that the lack of bronchial perfusion in IPL does not necessarily slow absorption in this region, lending further evidence that IPL successfully mimics the *in vivo* condition.

The absorptive surface area in the tracheobronchial region is much smaller than in the alveolar region, which is why simulated absorption from the tracheobronchial region was significantly slower than the simulated absorption from the alveolar region for the studied drugs (Table II). The difference in absorption between the two lung regions was even greater for the simulations using *in vitro* input parameters, because the pulmonary *P*_eff_ in that case was scaled with the thickness of the epithelium (Table II).

The *in vitro P*_eff_ value was calculated from a correlation between human intestinal *P*_eff_ and Caco-2 *P*_app_, which has often been used for biopharmaceutics intestinal absorption prediction, while the *ex vivo P*_eff_ value was estimated from IPL data using the IPL PBB model (23). The two methods used to obtain a *P*_eff_ value rendered significantly different values, especially when comparing the values for the alveolar region. The effect of this difference in *P*_eff_ value between simulations using *in vitro* or *ex vivo* input parameter could be seen for the alveolar absorption of solutions, which was much higher for the *in vitro* simulations (Fig. 4). However, the high *in vitro P*_eff_ value was compensated by a high tissue retention for salmeterol, resulting in a slower overall absorption rate. Similarly, *P*_eff_ would have a low impact on the overall absorption rate if dissolution is the rate-limiting step, which is the case for low solubility APIs. The difference in *P*_eff_ was smaller in the tracheobronchial region because of the scaling of the value in the *in vitro* simulations. Scaling of *P*_eff_ value between the lung regions was attempted in the IPL PBB model but this revised model was not able to explain the experimental data obtained from IPL (data not shown). In the simulations using *in vitro* input parameters, both salbutamol and salmeterol were absorption limited by retention in the lung tissue in the tracheobronchial region despite a big difference in *f*_u,tissue_ between the two APIs. This indicates that the permeability on the basolateral side of the membrane (*i.e*., transport from the membrane into the bloodstream) could be rate-limiting for APIs with low permeability and high *f*_u,tissue_. The effect of the permeability value on the overall absorption rate of solutions can be seen when comparing the *ex vivo* simulation of AZD5423 to the simulations of salbutamol and salmeterol. AZD5423, with a higher *P*_eff_ value, has a higher absorption rate in both lung regions compared with salbutamol and salmeterol.

The setting for tissue retention differed between simulations using *ex vivo* and *in vitro* input parameters because of the nature of the experimental data. Dynamic distribution was applied for the *ex vivo* simulations and equilibrium distribution was applied for *in vitro* simulations. When lung tissue distribution is fast, as for example with AZD5423, no significant difference between the two settings was observed. In contrast, for APIs like salbutamol with slow distribution, significant differences between the settings will occur (data not shown). For example, if an equilibrium distribution is applied, salbutamol will be highly retained in the tissue because *f*_u,tissue_ is very low, while if a dynamic distribution is applied, retention will be low because the rate of distribution is low (Table II). Predictiveness may be improved by measuring dynamic distribution in lung slices rather than equilibrium distribution, which will also better represent the actual absorption process (11,24,25).

The solubility used in the *in vitro* simulations was lower than in the *ex vivo* simulations, which resulted in a higher amount of API remaining as solid in the *in vitro* simulations (Fig. 4, Table III). In the *in vitro* simulations of APIs administered as suspensions (where dissolution was the rate-limiting step), the applied solubility seemed to be too low, as indicated by the low values for *C*_max_ and AUC. Solubility in phosphate buffer pH 7.4 might not represent solubility in lung lining fluid, and a more optimal input value for solubility needs to be found if simulations and models are to be improved.

The systematic exposure predictions in Fig. 2 based on the *ex vivo* input parameters performed well in predicting *in vivo* lung absorption, although they have *ex vivo* model origin. However, predicted and observed values were not identical, and additional investigation is required. One possible source of deviation is that the IPL and *in vivo* models used different administration techniques and inhalation devices, which would result in different deposition patterns in the lungs, which in turn would likely affect absorption (26). The deposition pattern was an approximate estimation for both the *in vivo* and IPL data, and so simulations using different deposition patterns were also performed to test the effect on simulated absorption. Deposition pattern did affect the plasma concentration, especially *C*_max_, and clearly, it is important to have a good estimate of deposition pattern; however, there is little experimental data to validate any given pattern (27).

Another possible source of difference is that the API batches were not identical for IPL and *in vivo* experiments, which could affect the dissolution rates. Differences in particle size distribution were accounted for in the simulations, but differences in other factors like shape and agglomeration behavior could not be accounted for because there was no available data on these factors. Another potentially important difference is that the lungs in IPL are excised from the rat body and are therefore composed of dying tissue. This tissue has been shown to be viable for several hours, but the loss in viability over time might alter absorption compared with the living tissue *in vivo* (28). Finally, it is difficult to simulate and predict *in vivo* plasma concentration-time profiles when the *in vivo* data are obtained from nose-only inhalation experiments, because the lung-delivered dose can only be an approximate estimate. Both the simulations using *ex vivo* and *in vitro* input parameters had lower *C*_max_ and AUC values than the *in vivo* data for two out of three of the APIs administered with nose-only inhalation, which suggests that the dosage applied in the simulations might be too low.

## CONCLUSIONS

This study has further demonstrated the usefulness of data obtained with IPL by showing that absorption parameters obtained by this method yield better predictions of rat *in vivo* lung absorption of both solution and suspension formulations than absorption parameters determined from standard *in vitro* measurements. It would be advantageous to use predictions based on IPL data during drug development in order to increase mechanistic understanding of the pulmonary drug absorption processes and to better predict how changes in drug substance properties and formulation will affect the *in vivo* performance of inhalation compounds.
